# An update on the incidence of dengue gaining strength in Saudi Arabia and current control approaches for its vector mosquito

**DOI:** 10.1186/1756-3305-7-258

**Published:** 2014-06-03

**Authors:** Al Thabiani Aziz, Salman Abdo Al-Shami, Jazem A Mahyoub, Mesed Hatabbi, Abu Hassan Ahmad, Che Salmah Md Rawi

**Affiliations:** 1 Department of Biology, Faculty of Science, University of Tabuk, Tabuk 71491, Saudi Arabia; 2 Department of Biological Sciences, Faculty of Science, King Abdulaziz University, P.O. Box, 80203, Jeddah 21589, Saudi Arabia; 3 Ministry of Health, Makkah, Kingdom of Saudi Arabia; 4 School of Biological Sciences, Universiti Sains Malaysia, Penang 11800, Malaysia

**Keywords:** KSA, Dengue, Mosquito, Control strategies, *Aedes eagypti*

## Abstract

**Background:**

The cases of dengue reported earlier in the late 1990s from the Kingdom of Saudi Arabia (KSA) occurred in the cities of Jeddah and Makkah. Although the kingdom has ample financial resources to establish effective control measures for the dengue vector, numerous cases of dengue occur and fluctuate in numbers from year to year. This necessitates a serious review of the current vector control strategies being practiced in order to identify the existing shortcomings. This short report provides an update on epidemiology of dengue in KSA (specifically in cities of Jeddah and Makkah) with a critical look at the current vector control strategies.

**Findings:**

In 2013, 4411 cases of dengue were reported, with 8 cases of mortality. This number of dengue incidence was four times higher compared to 2012. In 2013, the highest number of 1272 dengue cases was reported in May, while the lowest number (37) of cases was reported in September.

**Conclusions:**

It is evident that the control strategies of the dengue vector presently employed are inadequate. There seems to be serious deficiencies in following proper scientific procedures during field application(s) of control materials against the vector as is evident by the increases in the number of dengue cases as well as frequent outbreaks of the vector mosquito populations. In this review, some specific suggestions are made to draw attention to the relevant KSA authorities of the possible reasons behind unsuccessful control results and as to how to improve the strategy of dengue vector control in the kingdom.

## Background

During the last two decades, the epidemic of dengue with sporadic incidence of dengue hemorrhagic fever (DHF) has occurred in the cities of Jeddah and Makkah, Kingdom of Saudi Arabia (KSA) [1]. Furthermore, the onset and outbreak of dengue was also reported in the neighboring country, Yemen [2-4]. As a result, relevant Saudi health authorities became alert in this area and considerable attention was dedicated to controlling the vectors [5]. In terms of geographical location, KSA is situated in the south-eastern part of Asia. It has a long coastal boarder with different countries in Africa (Sudan and Egypt) and Asia. This puts the KSA near to the hotspot areas of epidemic diseases including dengue.

In KSA, three dengue virus serotypes (DEN-1, DEN-2, and DEN-3) were first detected in Jeddah in 1994 [6]. According to Mahyoub [7] the dengue virus was detected only in female *Aedes aegypti* mosquitoes. This indicated that *Ae. aegypti* is the main vector of dengue in KSA. In this country, there has been a sharp increase in the distribution of *Ae. aegypti*. El-Badry and Al-Ali [8] reported the recent establishment of viable populations of dengue vector inside and around homes in Al-Madinah Al-Munawwarah (~600 km far from the relatively hot zone of Jeddah and Makkah), where this mosquito was previously absent. According to an estimate, the number of Muslim pilgrims to Makkah and its surrounding areas is expected to rise to nearly 12 to 17 million by 2025 [9]. As of 2010, an estimated number of 2.5 million Muslims visited the holy City [9]. Consequently, large influxes of visitors from potentially dengue-endemic areas, and the presence of ecological features conducive to the spread of *Ae. aegypti,* such as water storage habit, uncovered domestic water storage, warm climate and well-developed transportation networks in the country are of crucial significance in terms of public health issues. Despite all this, there is no serious concern shown by the relevant health authorities on the possibility of large-scale dengue outbreak(s). Although there is likelihood of outbreak and spread of dengue in epidemic proportions and rise of its vector populations, relatively less attention is paid on updates concerning the outbreaks and epidemiology of dengue in the Middle East, especially in Saudi Arabia and Yemen [3]. Therefore, the aim of this short report is to briefly update the recent records of dengue and the current efforts employed to control the dengue vector in KSA. It is hoped that this report will help researchers and mosquito control managers in the region to develop new techniques or strategies for improving current control efforts. It is understood that developing effective control strategies of the dengue vector might be very challenging as some *unconfirmed* reports about spread of mosquito vectors to the northern part of Saudi Arabia (cities of Tabuk and Amlog) have emerged for the first time. Additionally, the dengue virus has appeared with new serotypes and this makes the dengue control problem more challenging and perhaps an endless battle against the disease and its vector mosquito.

## Findings

**Figure 1:**
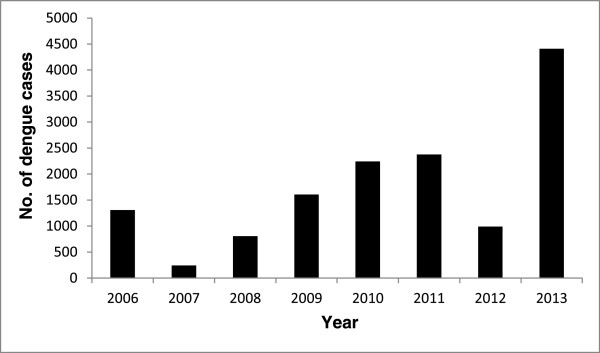
Number of confirmed dengue cases reported from 2006 to 2013 in cities of Jeddah and Makkah, Kingdom of Saudi Arabia.

**Figure 2:**
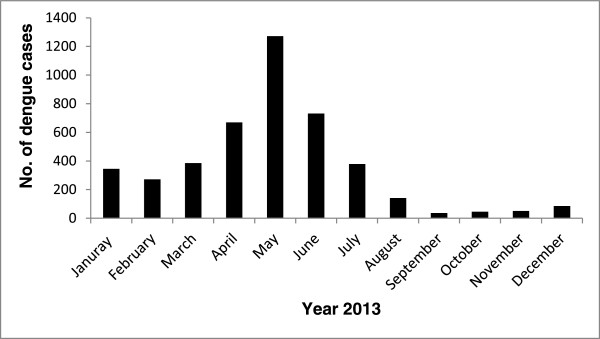
**Reported cases of dengue by the month in 2013 in the cities of Jeddah and Makkah, Kingdom of Saudi Arabia**.

Information provided by the Saudi Ministry of Health (MOH) in 2014 on the number of reported dengue cases in Jeddah and Makkah from 2006 to 2013 is presented in Figure 1. It can be seen from the figure that the highest number of cases was reported in 2013 (4411), followed by 2011 (2376). In 2013, the highest number of cases (1272) was reported in the month of May, while the lowest number (37) of cases occurred in September (Figure 2). In 2013, out of a total of 4411 reported cases, 3108 cases occurred among people in 15 to 44 years age range; of these, 79.23% of the patients were male. On the other hand, the percent of local Saudi people who were diagnosed with dengue was 38% while the rest were foreign migrant workers in the country.

## Discussion

### Physical and cultural strategies for dengue vector control in Saudi Arabia

These strategies for controlling the dengue vector can be partially effective for preliminary elimination of the vector mosquito and primarily include elimination of the possible breeding sites of larvae. However, such efforts are rarely practiced in KSA. Also, public awareness of life cycle of the dengue vector and its favorable domestic and peri-domestic habitats is minimal. Therefore, it is suggested that intensive efforts should be made to enhance public awareness for the overall health promotion of the general public in KSA. Association between larval control strategies and health education of the public should be maintained. The residents should be aware of not only the control activities conducted by the special mosquito control teams but should also seriously respond to the health education-related information dissemination scheduled by the relevant governmental authorities. For dengue control, public awareness and education concerning the breeding sites of the dengue vector mosquito and its physical and cultural control are vital in population management of this container-breeding vector.

### Chemical control

Generally, control of dengue vectors in the Middle Eastern region is primitive and in most cases non-existent [7]. In KSA, during the last few decades, several insecticides have been employed intensively for controlling the dengue vector. For example, pyrethroids, such as cyfluthrin, deltamethrin, lambdacyhalothrin, permethrin were reported to be effective chemical controls (adulticidal) agents in KSA [10]. These insecticides are well-known for their quick and efficient knock-down action, this property has made them the most popular chemicals for insect control including mosquitoes [11,12]; however, frequent and regular use of these insecticides is conducive to the development of resistance in the target insect(s) globally [12].

Based on a municipality report of the city of Makkah, in 2007, the estimated amount of undiluted mosquito adulticides and larvicides used were 17975 and 3899 liters, respectively [13]. In early 2006, the KSA government substantially increased mosquito control budget to prevent the spread of dengue in Jeddah; the estimated budget amounted to seven billion Saudi Riyals [10]. However, even with such a high expenditure, no significant decline in the dengue incidence occurred [14] and spatial distribution of *Ae. aegypti*[8] as well as circulation of the four dengue serotypes [15] persisted. In view of such costly control efforts, it is assumed that this unexpected failure could partially be due to contracting out the mosquito control work to private companies that lacked in terms of precise mosquito control knowledge, use of appropriate science-based strategies, and perhaps efficient professional supervision. It is suggested that public awareness concerning appropriate measures of hygiene and sanitation to eliminate the possible breeding sites of mosquitoes should be organized frequently by respective local municipal and federal Ministry of Health authorities. In addition, mosquito vector control by house-to-house spraying in KSA is still not practiced properly because it is managed by private companies having small teams of mostly untrained or undertrained staff that apply the insecticides without much knowledge and experience of insecticidal application and dealing with insecticides and mosquito population management. In many cases, the control resulting from contracted private companies was inadequate and ineffective. This may have been due to insecticidal under dosing by the contractors to reduce their insecticidal, operational, and management costs. In addition to all of the above-mentioned shortcomings, it has been observed that, the workers sometimes mixed and applied the insecticides merely by themselves without any expert supervision concerning dosage. Consequently, significant negative impact on control effectiveness would be expected. Furthermore, there are some other factors that have some negative bearing on control effectiveness of the dengue vector in KSA. For example, several entrepreneurs in Jeddah were reported to promote adulterated insecticides as a "magic fix" to kill mosquitoes without any awareness of their side effects and long-term adverse impact on human health and possible development of resistance in the target mosquitoes [16]. It would be fitting to emphasize here that field applications of insecticides under contracts are made on a routine basis without any pre- and post-treatment population assessment(s) of prevailing populations of mosquitoes, and thus unnecessarily amplifying insecticidal pollution in the environment as well as risking onset of resistance phenomenon in the target mosquitoes. In Saudi Arabia, infrastructure development and urbanization along with human population growth have been rather rapid during the past 40 years or so. Specifically, the city of Makkah is developing rather rapidly in various sectors without any consideration or planning to curb the spread of dengue vector. Nevertheless, for any dengue-related entomological observations, permission to enter people’s dwellings must be granted by the male head of the family (cultural practice) and is often an impediment in the crucial requirement of mosquito population assessment and dengue control process. Some residents are afraid of police or immigration authorities and therefore entry of mosquito researchers and spray teams into homes for research and control purposes becomes more difficult and this problem is usually overcome by following educational health officers for the relatively easier access to the houses.

## Competing interests

The authors declare that they have no competing interests.

## Authors’ contributions

All authors contributed equally to this work. All authors read and approved the final version of the manuscript.
